# From Physical to Spiritual: A Qualitative Study of Jakartans Health & Sickness

**DOI:** 10.3390/ijerph16193564

**Published:** 2019-09-24

**Authors:** Yasinta Astin Sokang, Alvin Henry Westmaas, Gerjo Kok

**Affiliations:** 1 Department of Work and Social Psychology, Maastricht University, 6229 ER Maastricht, The Netherlands; alvin.westmaas@maastrichtuniversity.nl (A.H.W.); g.kok@maastrichtuniversity.nl (G.K.); 2 Faculty of Psychology, Krida Wacana Cristian University, Jakarta 11470, Indonesia; 3 Faculty of Health Sciences, University of Applied Sciences, 2333 CK Leiden, The Netherlands

**Keywords:** perception, Jakarta, health, sickness, qualitative study

## Abstract

Understanding the perceptions of health and sickness can help the government, health providers and health promoters encourage individuals to participate in healthy behaviors and to follow a healthy lifestyle. Jakarta, the capital city of Indonesia, is a culturally, socially and financially diverse city, with complex health care needs. As yet, there is no published data available about Jakartans’ (i.e., the citizens of Jakarta city) perceptions on health and sickness. This study aimed to describe what health and sickness mean to Jakartans. To this means, we collected data using an open-ended survey about the meanings of health and sickness from 640 Jakartans. Five main themes of health and sickness emerged. The five themes of health were health as a physical condition, a psychological condition, a spiritual condition, a capability to carry out daily activities, and a healthy lifestyle. The themes regarding sickness were sickness as a physical condition, a psychological condition, an abnormal circumstance or bad situations, a spiritual condition, and an inability to carry out daily activities. We discussed how the above-mentioned perceptions might influence the daily health-related behaviors of Jakartans. In contrast to the typical biomedical approach, we found that, in Jakarta, health was not merely seen as a causal effect of the physical world. Further details are discussed.

## 1. Introduction

In Indonesia, improving the health condition has been a continuous effort [1]. For example, the number of health facilities has increased each year from 9.665 units in 2013 to 9.731 units in 2014 [2]. However, these health facilities are often unable to provide optimal health supports due to the limitation of human resources [1]. Moreover, providing health services by increasing the number of health facilities requires a significant investment in terms of finances and time [3]. Therefore, it has been argued that in a highly-populated country such as Indonesia, preventive health care—for example, health promotion programs—is a better way to improve health quality as compared to curative approaches such as increasing the number of health facilities [4].

In order to implement an effective health promotion, it is important to understand health in its cultural context. People’s perceptions about health influence their actions—for example, the behaviors they participate in and whether they have a healthy lifestyle [5]. It has been shown in studies that patient perceptions of their current health problems in part determine whether (and how) they would use health care services [6,7,8]. A better understanding of the cultural context of health perceptions can help health care providers to provide the type of health care that meets the needs of the citizens, to improve the communication between health care providers and patients, and to improve patient satisfaction [5,9].

Indonesians tend to interpret health not only in term of the physical condition but also in terms of other aspects. These aspects include art, which is defined by the chance to enrich and express one’s self [10]; religiousness, which defines health as a gift from God, a blessing [11]; an uncomfortable feeling, which is defined by being unable to work, losing appetite, and not having any money (‘kantong kering’) [12].

Although many studies on the perception of health have been conducted in various geographic areas of Indonesia, there has been (to our knowledge) no international, peer reviewed publication on the perceptions of health and sickness of Indonesian; let alone a publication on the perceptions of health and sickness of the citizens of Jakarta (henceforth will be called “Jakartans”), despite of the fact that Jakarta is the most multicultural city in Indonesia. As the capital, Jakarta is the most populated city in Indonesia [13]. This dense population brings out multiple problems such as poor drainage systems and waste problems, which cause frequent flooding [14]. Such problems are detrimental both financial- and health-wise. As a consequence, health care needs have been increasing enormously [1]. Given the aforementioned benefits of preventive health care, an ideal solution would be to provide health care by means of public health promotion.

As discussed above, it is important to provide an effective health promotion that approaches the issues not only theoretically but also in a culturally sensitive way. Intervention Mapping (IM) is a health program planning approach that is based on using theory and evidence as the foundations for an ecological approach to assess and intervene health problems and to engender community participation [15]. One of IM’s key points is that it provides clear descriptions to guide the process of planning and designing, developing, implementing, and evaluating the program objectives, supported by theory and empirical-evidence [16,17]. IM consists of six steps with several tasks at each step.

In order to provide an effective health promotion, as an initial step, this study focused on the Step 1 of IM. Step 1 of IM highlights the importance of assessment to understand the behavior and environmental determinant, as well as to understand the characteristics of the community, including the social perspective of a community at risk for health-related issues. Therefore, the purpose of this study is to describe what health and sickness actually mean to Jakartans. This information can be used by the government, health care providers, health promoters, and nurses to ensure that health care services not only provide good care, but also to meet the specific needs of the inhabitants of Jakarta.

## 2. Materials and Methods

The qualitative descriptive approach was used to capture the participants’ perception of health and sickness. The approach was used as it provides direct descriptions of the target phenomenon. The participants explain their experiences in their own words and presented the facts of the perception in everyday language [18,19,20]. This approach enables us to address the cultural values of health and sickness perception.

### 2.1. Settings and Respondents

The data were collected by means of an open-ended survey which was adapted from Liem & Yuniarti [11]. This survey comprises two statements which designed to assess the respondents’ perceptions of health and sickness. Specifically, respondents were asked to complete the sentences ‘Health is…’ and ‘Sickness is…’ based on their own perceptions. This sentence completion was chosen to provide the opportunities to respondents to use their own words to explain their perceptions [21]. The flexibility of this approach also allowed respondents to make a wide variety of responses, which in turn provided us a rich dataset. The data were collected in multiple rounds. At the end of each round, an initial analysis was conducted to identify the emerging themes. Data collection was stopped when the saturation of the data was achieved; no new themes emerged from the data [22,23]. The raw data supporting the conclusions of this manuscript will be made available by the corresponding author, without undue reservation, to any qualified researcher.

The study was conducted by the first author in Jakarta. With a total area of 1324.66 km^2^, and an estimated population of 10 million people in 2015 [24], it is the most highly populated city in Indonesia. The inhabitants come from different areas and ethnic backgrounds, and they bring their own culture and values to the city [25]. Respondents in this study were recruited from September 2014 until November 2014 in all areas of Jakarta. Respondents were included in the study if they were born in or had residence in Jakarta and were of age 12 or older. The respondents were recruited using a convenience sampling.

Five research assistants were trained by the first author to collect the data and to answer any questions potential respondents might have. Upon finishing their training, the research assistants went to public areas such as shopping malls, markets, streets, hospitals, and schools in order to find potential respondents. Each potential respondent was briefly informed about the purpose, procedures, and confidentiality of the study. After respondents had agreed to participate by providing an oral consent, they received a survey package containing the questionnaire, a pen and a clipboard. The questionnaire included a written consent, information about the purpose and confidentiality of the study, and information about members of the research team (name, faculty, university, and email address). For respondents who are underage (under 16 years old (y/o)), the written consent was given by the parents, or the school principle and/or teacher. Together, written informed consent was obtained from all adult participants and the parents/teachers of non-adult participants. The respondents were instructed that there were no wrong answers, and they should complete the sentences based on what first came into their mind. In addition to completing the two open-ended questions, respondents filled in four demographic questions about sex, age, education level, and income/allowance per month. After the surveys were completed, respondents were asked if they had any questions and thanked for their participation.

The study was approved by both the Ethical Review Committee of the School of Psychology and Neuroscience, Maastricht University (Reference number of research line: ECP-04-09-2012), and an internal peer reviewer of the Center for Research and Community Service of Krida Wacana Christian University, Jakarta (Contract number: 04 /UKKW/LPPM-FPSI/Lit/I/2014). The written informed consent was obtained from all adult participants and the parents/teachers of non-adult (under 16y/o) participants.

### 2.2. Analysis

All data were analyzed using content analysis with an inductive approach. This approach was used because there had been little to no existing information about the meaning of health and sickness from the perception of Jakartans. The analysis process included open coding, creation of categories, and abstraction [26]. In the open coding part, the researchers applied human coding for this study and used the Indonesian Dictionary to compare the meaning of the words. The open coding and categorization were carried out by two teams of three researchers. Each team conducted the open coding of all data by means of discussion within the team. The coding rules are described in more details in the Appendix A, Appendix B, and Appendix C.

In the creation of categories part, the coded words were categorized in groups of similar statements. Subcategories with a similar word content across the teams were grouped into categories [27]. If a statement did not fit an existing group, a new group was formed. Lastly, in the abstraction part, the resulting groups were discussed between teams in order to create themes. The categories with a similar theme were grouped into main themes and named using words that respondents had generated [28].

## 3. Results

In total, 640 Jakartans (51% females) Jakartans completed the study. There were no data dropped out as the participants filled the sentences completely. Potential participants who refuse to participate mainly because they were in a rush or had no time to join the study. The sample has a mean age of M = 23.22 (range: 12–60 years). Although respondents from all educational levels participated, people with a senior high school education were over-represented (see Table 1). The demographic distributions correspond to that of the research assistants, which might explain the overrepresentation. Although the instruction was to look for respondents of various ages, the research assistants´ age might influence the reactions given by the prospective respondents.

We categorized the meanings of both health and sickness in five main themes (see Table 2 and Table 3). Each main theme will be described with regard to the ‘single term’ and the ‘in combination’ categories. In the ‘single term’ category, we included statements that can only be categorized into one single theme. Often times, participants provide a statement which can be categorized into multiple themes. These statements contain meanings which overlap across multiple themes. Therefore, we grouped the statements into two or more categories under the ‘in combination’ category. We will only describe themes that more than 30 respondents referred to and we did not consider the statements from fewer than 30 respondents since these may be unrepresentative of the population. Participant's statements for their perception of health were coded with H (e.g., H1, H2, etc.) and their statements for their perception of sickness were coded with S (e.g., S12, S13, etc.). The results were ordered from the most to the least frequently mentioned theme.

### 3.1. The Meaning of Health

#### 3.1.1. Health as a Physical Condition

About 23.8% of the respondents perceived their health solely in term of a physical condition (single-term category). Health was identified as the optimal functioning of the organs and the immune system, fitness, strength, and as being physically safe and sound.

*‘Having a physical condition—including a good immune system—which can fight off disease and bacteria’ (female, 18 years old)*

*‘Having strength and a strong immune system which help us stay healthy. Health is related to the body organs (male, 22 years old)*

Almost half of the responses indicated health as a physical state in combination with other condition. According to the respondents, health could be interpreted as a combination of the physical condition and the psychological condition *[H1] ‘being psychologically and physically OK. Psychologically: feeling no stress, not having many things on your mind, not being dominated by negative thinking. Physically: having a lot of energy, not easily getting ill, having passion and not being dull’ (male, 20 years old);* spiritual *[H2] ‘a physical condition which is free from pain or diseases, healthy both physically and spiritually’ (female, 18 years old)*; social *[H3] ‘being physically free of pain or weaknesses, so one can interact socially and to easily adapt’ (female, 18 years old,)*; and a capability to carry out daily activities *[H4] ‘when someone physically has the energy or stamina to carry out activities in a normal way’ (female, 20 years old)*.

In addition to perceiving health as a combination of the physical condition with one other condition, some respondents conceptualized health as a combination of physical condition with more than one condition—for example, they perceived health as a combination of physical condition, psychological condition and having a healthy lifestyle *[H5] ‘[health is] about a good physical and spiritual condition because one has a regular lifestyle and discipline’ (male, 22 years old)*; or the combination of many different conditions *[H6] ‘being psychologically, mentally, physically and socially healthy, so that daily activities are not disrupted’ (female, 18 years old)*; as well as the absence of physical, psychological, and economic disorders *[H7] ‘a condition in which someone does not experience any physical, psychological, or economical disorder’ (male, 19 years old).*

*[H8] ‘A state in which the body and the soul are in a normal condition, and there are no limitations on performing activities’ (male, 30 years old)*

*[H9] ‘Free from pain and physical weaknesses. When you can socialize, adapt, and have a good personality and state of mind’ (male, 18 years old)*

*[H10] ‘A condition when we feel happiness both physically and spiritually; and to feel comfortable to do anything, without any interference from the inside [e.g., physical constraints]’ (female, 19 years old)*

#### 3.1.2. Health as a Psychological Condition

Respondents perceived health as a psychological condition, which was perceived as a combination of calmness, peace, and joy in heart, mind, and soul— even in attitude. It was expressed as a pleasant and soothing feeling and could be interpreted as a clear and unencumbered state of mind.

*‘Whenever we feel jubilant, comfortable and safe, as well as in a fresh condition’ (female, 23 years old)*

*‘Live in peace and unencumbered by problems (female, 25 years old)’*

Slightly fewer than a third of the respondents considered health as a psychological condition that was related to one other condition: physical (see H1; H5; H8; H9), spiritual (see H6), social (see H9), the ability to carry out daily activities (see H8), and a healthy lifestyle (see H5). The psychological condition was also included in combinations of many conditions (see H6), e.g., health as the absence of physical, psychological, and economic disorder (see H7). In addition, some respondents stressed the combination of psychological conditions and the ability to perform activities.

*‘Can perform various activities with a rational mind’ (male, 26 years old)*

*[H11] ‘Comfortable, peaceful, stable condition so that someone can carry out daily activities well’ (male, 24 years old)*

#### 3.1.3. Health as a Spiritual Condition

Respondents also defined health as a reference to their spiritual values. Health was identified as a grace and a blessing. Some respondents pointed out that health cannot be replaced by anything, and it was seen as a blessing to be continually grateful for.

*‘Something that cannot be replaced by anything and something that we should be continually grateful for, because being healthy is a blessing in life’ (male, 29 years old)*

Health was also described as a combination of spirituality with one or more other condition, such as the physical condition (see H2; H10), the psychological condition (see H6), the ability to carry out activities (see H10), or a combination these conditions (see H6).

#### 3.1.4. Health as a Capability to Carry out Daily Activities

Fifty respondents (7.8%) perceived health in terms of the ability to carry out activities without being distracted.

*‘Being able to do something with [full] capacity and to the best of one’s abilities’ (female, 23 years old)*

This theme was also mentioned in combination with other conditions, such as physical (see H4; H8; H10), spiritual (see H10), and psychological (see H11) conditions.

#### 3.1.5. Health as a Healthy Lifestyle

A lifestyle was perceived to be healthy in cases in which it includes a healthy diet, an exercise, and a clean environment.

*‘[to] Maintain cleanliness, [to] exercise, [to] eat regularly and drink plenty of water’ (male, 26 years old)*

*‘The key to being healthy is hygiene’ (female, 40 years old)*

The respondents also reported that health was perceived as a combination of physical and psychological components, as well as a healthy lifestyle (see H5).

### 3.2. The Meaning of Sickness

#### 3.2.1. Sickness as a Physical Condition

About 26.3% of the respondents perceived sickness as a physical condition. Sickness was defined as a weak physical condition or the non-functioning of certain body parts due to a decrease in the immune system because of the presence of a disease or virus.

*‘Weak body and [to feel] unfit or drastically decreased-stamina’ (female, 18 years old)*

*‘[the] Condition of disorder or dysfunction of the body or a body part’ (male, 32 years old)*

Furthermore, two-fifths of the respondents perceived their own sickness in combination of a physical condition and other conditions, including psychological *[S12] ‘the condition of a physical, mental or disturbed mind or having a (psychological) disability’ (male, 25 years old*); spiritual *[S13] ‘physically, when we are injured. Inwardly, when we have a lot of thoughts and cannot find a way out’ (female, 25 years old)*; social *[S14] ‘one’s condition when one is exposed to disease and disability, both physically and socially’ (female, 20 years old)*; an inability to carry out daily activities *[S15] ‘the circumstances in which the body is not in a good or prime condition because of a disease or germs from a particular virus, so that the body becomes weak and this hampers everyday activities’ (female, 21 years old)*; and an unhealthy lifestyle [S16] *‘the state of the body affected by disease and followed by a dirty and nasty environment’ (male, 19 years old)*.

Some respondents also conceptualized sickness as physical condition combined with two or more other conditions of sickness at the same time, for example, a combination of physical, psychological, and spiritual conditions *[S17] ‘someone who does not have a passion for life. Health or sickness is not always because of a physical factor, because to be human [one] has [the] elements of [the] body, [the] soul and [the] spirit. Then, if one of these [elements] goes wrong, a person will become sick’ (female, 23 years old)*; or physical, psychological, and an inability to carry out daily activities *[S18] ‘there are parts of the body that are ill or disturbed or in a bad condition, [one] cannot move freely or think; the [psychological] heart is often unhappy or stressed’ (female, 57 years old)*; physical, psychological, and social *[S19] ‘[the] circumstances when someone needs others to help cure the illness [physical and mental]’ (male, 18 years old)*; and a combination of many conditions *[S20] ‘the circumstances in which a person experiences a physical disorder, or psychological or economic problems, or all of these’ (male, 19 years old)*.

#### 3.2.2. Sickness as a Psychological Condition

Sickness was also seen as a psychological condition. Respondents expressed the notion that someone could become sick whenever one experiences stressful situations, such as unsolved problems, or some limitations on being able to exert one’s free will. These stressful situations can cause discomfort and sad feelings, as well as loss of the spirit to live.

*‘Sick and healthy people are actually controlled by the mind, a healthy person will feel ill if his mind considers himself sick’ (male, 19 years old)*

More than a quarter of the respondents interpreted their sickness as a psychological condition in relation to other conditions such as a physical condition (see S12), a spiritual condition (see S17), and an inability to carry out daily activities (see S18). Respondents also conceptualized sickness as a combination of a psychological condition and an inability to perform activities.

*[S21] ‘Uncomfortable or unusual conditions so that the person feels uncomfortable or has problems in performing daily activities’ (male, 24 years old)*

*[S22] ‘Cannot think properly and cannot live a good life’ (female, 20 years old)*

#### 3.2.3. Sickness as an Abnormal Circumstances and Bad Situations

Respondents regarded this particular theme as a single-term category; they did not perceive sickness as a combination of abnormal and bad situations with other situations or conditions.

*‘Abnormal, out of the ordinary conditions, below the normal line’ (male, 39 years old)*

#### 3.2.4. Sickness as a Spiritual Condition

Some respondents described sickness as a temptation and a test, as someone staying away from God. From this point of view, a sickness exists in order to keep humans close to God.

*‘One result of human negligence and one of the means of allowing humans to be closer to God’ (female, 32 years old)*

Respondents also identified sickness as a combination of a spiritual condition with other conditions, such as physical (see S13) and psychological (see S17).

#### 3.2.5. Sickness as an Inability to Carry out Daily Activities

Respondents stressed the meaning of sickness as a person’s inability to perform activities. The inconvenience related to the inability to carry out daily activities was also interpreted as sickness. Although this was seen as a natural part of life, sickness was also understood as the inability to achieve maximum results when one is working on something.

*‘Cannot do [any] activities’ (female, 28 years old)*

*‘When I cannot achieve the maximum in every way’ (male, 19 years old)*

Sickness was also seen as an inability to carry out daily activities in relation to both physical (see S15, S18) and psychological conditions (see S18, S21; S22).

## 4. Discussion

The aim of the present study was to explore the perceptions of health and sickness among Jakartans. A more comprehensive understanding of health and sickness will shed light on the cultural context of health and sickness that influence certain actions, such as whether or not one would adopt a healthy behavior or a healthy lifestyle. This information can help health care providers to provide services which are in line with the public understanding of health and sickness. Overall, our findings demonstrate that respondents perceived health and sickness not only as a physical condition, but also in a combination of many other conditions. Simultaneously, we also found that some respondents identified their health and sickness in one-single condition unconnected to other conditions of health or sickness (see Appendix A).

In this study, health was seen as a positive term. Health was perceived in terms of fitness and bodily strength (physical), the calmness of the mind and the soul (psychological), a close relationship with God (spiritual), and the ability to be active or productive (capability to carry out daily activities). Some respondents described a healthy condition as a result of a healthy lifestyle which includes the discipline in regards to maintain a healthy diet and to carry out a physical activity. Other respondents perceived health as a condition that allowed people to do multiple activities. These findings were in line with a previous study from Hidajat [10] in East Java, Jogjakarta, and Bali regions which showed that people from East Java and Jogjakarta perceived their health and sickness from Javanese philosophy and attitude of life. In the Balinese society, for instance, health and sickness were related to the opportunity to pour out one’s art expression and self-improvement values. Our results were also similar to those of previous research in which health has been interpreted as a positive attribute—or example, as being well-functioning, having a good physical condition, a positive state of well-being, a positive feeling, and an ability to carry out desired or required activities [29].

Our result showed that respondents perceived health as an optimal physical condition. This physical condition of health was also linked to other conditions, such as psychological and spiritual conditions of health, the capability to carry out daily activities, and to have a healthy lifestyle. These conditions were mentioned in isolation or as having a causal effect on another condition. For example, health was interpreted as a healthy bodily condition that enables someone to perform various daily activities. So, for this example, healthy conditions cannot be interpreted as only a physical condition, but also as the ability to perform daily activities. This result was similar to findings from previous studies about health, which either investigated the meaning of health from the perception of the general population or some specific subgroup [30,31,32,33]. For instance, Mendelson [31] showed that Mexican American women perceived health as a compilation of a good physical health, a sound mental health, and a socially and spiritually satisfying life. The study of Isaak & Marchessault [33] on youth and adults of an Aboriginal community in a Northern Manitoba First Nations Community also defined health as various combinations of: changes in diet and activity, positive adult role models, traditional practices, and the significance of making good choices.

Various studies have stressed the finding that health can be both directly and indirectly influenced by social, economic and environmental circumstances [34,35,36]. This perception was also reflected in the daily activities of Jakartans, who associated being ‘healthy’ with the ability to work, to perform daily activities, and to be productive. This finding might be related to the fact that most of the respondents of the present study were from the low-income group (below IDR three million per month), which is close to 65% of the Jakartans who works as laborers and who receive daily (as opposed to monthly) payments [37]. Based on their work hours in February 2016, 4.5 million people (89.83%) in Jakarta worked for 35 hours or more per week. Receiving daily payments also mean that whenever laborers take a sick leave, they do not get their daily wage [38]. This leads them to worry about the income reduction due to the unpaid sick leave and thus, consequently, made them to still work in spite of being sick. This situation is different to that in the Western countries, where laws that require a company to pay sick leave exist, for example, up to five days for employees who need to recover from the flu [39,40,41].

A healthy lifestyle was identified as one condition of health by the respondents. Nowadays, in Jakarta, many people go to the gym, or take yoga, Zumba or Pilates classes [42]. At the same time, however, as long as they work out or go to the gym, people are less likely to care about what they eat, because they believe that they can eat whatever they want [43]. In contrast to this view, the results of the present study showed that respondents tended to emphasize a healthy/ unhealthy diet, and only a few of the respondents stressed the importance of exercise as part of health and sickness. This suggests that our respondents do not view health and having a healthy lifestyle in a comprehensive way. This limited perception of health may contribute to the high prevalence of cardiovascular disease, obesity and diabetes in Jakarta [44]. These diseases are mainly caused by an unhealthy lifestyle, particularly an unhealthy diet and a lack of exercise [42].

Importantly, health was also conceptualized in terms of the peace of mind or the psychological tranquility by our respondents. The psychological condition was described as a pleasant and soothing feeling, and as a clear and unencumbered state of mind. This perception may relate to the needs of the Jakartans for psychological care and recreational areas. Most leisure spots in Jakarta are in shopping malls, due to the lack of public space despite the government’s effort to build more urban open spaces [45]. Residents usually travel outside Jakarta, for example to Puncak, Bogor or Bandung area for recreational activities; however, they must navigate heavy traffics to reach these destinations [46].

The high demands and the fast-paced lifestyle in Jakarta are believed to contribute to high levels of stress in the community [47]. Nevertheless, psychological health services at the primary health level are limited, due to the low ratio of clinical psychologists compared to the Indonesian population [48]. Reports indicate that Indonesia has 500–600 clinical psychologists, whereas, Jakarta alone needs 341 clinical psychologists to meet the demands of the primary health services. This means that, only a few primary health services in Jakarta have a clinical psychologist as part of their team [49]. This is in stark contrast to psychological services provided in most primary health services in Jogjakarta and Sleman [48,50]. Clinical psychologists at primary health services in Jogjakarta provide psychological services on every working-day at a low cost (+USD 0.5) due to the financial supports from the government [51]. The availability of these services makes people more familiar with psychologists, more comfortable with the idea of consulting with them, and it reduces any negative stigma on psychological services [52].

While health was associated with positive terms in our study, our respondents perceive sickness in negative terms, such as weakness, inability, being infected with disease, feeling uncomfortable, and being cursed. Sickness was not interpreted simply as non-healthy conditions; instead, this conceptualization of sickness can be seen as more complex than the biomedical health approach [53], in which health and sickness are mostly seen as a result of, for example, sanitation and infection [5]. Here, sickness was defined as abnormal conditions and bad situations, when the result of things not happening as they should, or as something out of the ordinary.

Sickness, for the respondents, was associated with spirituality, for example. It was perceived to be trials by God and a test of one’s faith. Respondents tended to believe that sickness was a lesson taught from God to make them appreciate the life that had been freely given to them. People thus use prayers in the hope of healing their sickness, because there is a tendency to see everything spiritually and put it in God's hands for better results. This finding can be linked to research carried out in other countries which has investigated sickness as a reference to various conditions of spirituality. For example, the importance of praying, reading the Bible, attending church, or practicing traditional activities to remain in harmony with cultural values [31]

In a society where sickness is interpreted from a spiritual perspective, sickness also can be treated differently (as compared to the biomedical approach) [53,54]. People tend to go to traditional health practitioners, where they drink herbal regimens such as Indonesian herbal drinks (jamu) or Chinese herbs, or receive acupuncture and massages to heal their sicknesses [55,56,57]. Peltzer & Machleidt [58] found that people in developing-countries seek traditional medicines because they believe in the effectiveness of these medicines, sometimes in spite of the lack of evidence for their benefits. The high confidence they place in traditional medicines often means the people would try them first before they go to a physician [56].

Respondents in our study implied that there were sicknesses which result in the inability to get up or carry out daily activities. This means that someone is called sick if one is not productive or cannot perform daily activities. In this perspective, sickness cannot be equated with a physical condition because people tend to work despite being (clinically/physically) sick. The tendency to work despite being sick can also be linked to the spread of diseases as well as being related to income issues, as explained before. If someone still goes to work while being sick, there is a possibility that others will be infected, especially in a highly-populated city [59,60] such as Jakarta.

Our results have several implications. Firstly, there is a need to improve the health education and promotion systems to adapt to the fast-paced life in Jakarta. This information needs to catch the Jakartans’ attention and at the same time motivate them to implement it. The health education system can include the importance of a healthy lifestyle, including exercising alongside having a healthy diet. The information about how diseases spread should also be disclosed, so people would understand the risk of going to work despite being sick. Health promotion can be done by providing leisure spots in the city, for example, a playground with sports facilities, so that people may get to relax while they exercise. Secondly, the government needs to consider involving religious leaders in conducting health promotions. This suggestion arises from the fact that people perceive health and sickness from a religious perspective. The involvement of religious leaders might contribute to fulfilling the religious aspect of health, without putting aside the community’s health in general. Thirdly, the government needs to consider providing health care that facilitates typical laborers to get health checks. For example, to provide services after normal working hours and at the weekends. This suggestion might counter the financial issues for people who are paid on a daily basis. Lastly, given the finding that people tend to visit traditional health practitioners, the government should consider including traditional, complementary, and alternative medicine (or TCAM) in its health promotion and health intervention activities. A collaboration between modern medicine and evidence-based traditional or alternative medicines may help to provide a better health care service, one that is more in line with people’s needs, beliefs, and values. It could also be used to maintain and improve the health of particular communities.

Despite the study’s contribution, this research had several limitations. Firstly, the research findings may not be generalizable to other populations in Indonesia. As stated before, the cultural context influences people’s perceptions and experience. As Indonesia is a country with a high cultural diversity, each region in Indonesia has its own cultural characteristics. Additionally, to generalize our results to the Jakarta population better, it is necessary to investigate the perceptions of health and sickness of a larger sample of Jakartans with quantitative and statistical procedures. Secondly, as stated at the results section, the research assistant’s age might have an influence on the responsiveness of the prospective respondents (i.e., respondents with the similar age range with those of the assistants might be more inclined to participate in the study). Further research might consider recruiting research assistants with more diverse characteristics.

## 5. Conclusions

This study discovered that Jakartans perception about health can be grouped into five main categories: health as a physical condition, health as a psychological condition, health as a spiritual condition, health as a capability to carry out daily activities, and health as a healthy lifestyle. Meanwhile, sickness is perceived as a physical condition, a psychological condition, an abnormal circumstance and bad situations, a spiritual condition, and an inability to carry out daily activities. This multidimensional view has effects on Jakartans behavior towards health and sickness. For instance, this view may explain why some people keep working despite of being sick, and why some people use prayers in the hope of healing their sickness. Additionally, we also discussed that the current health system in Jakarta, which emphasizes on physical conditions and is heavily influenced by the biomedical perspective, may not accommodate this multidimensional view of health and sickness.

## Figures and Tables

**Table 1:** Respondent Characteristics.

Characteristic	*N*	(%)
Sex		
Male	313	48.91
Female	327	51.09
Age		
Mean (range)	23.22	(12–60)
12–15 years old	8	1.3
16–19 years old	268	41.9
20–30 years old	318	49.7
31–60 years old	46	7.2
Education		
Elementary	5	0.78
Junior High School	24	3.75
Senior High School	501	78.28
Diploma	13	2.03
College Graduate	91	14.22
Masters	6	0.94
Income/ allowance per month (IDR)		
<1 million	273	42.65
1–3 million	242	37.81
>3 million	125	19.54

**Table 2:** Themes of the Meaning of Health.

Main Themes	*N*	%	Subcategories	*N*	%
Single-Term			In Combination		
Physical	152	23.8	Physical and Psychological	99	15.5
			Physical and Spiritual	71	11.1
			Physical and Capability to carry out daily activities	44	6.9
			Physical, Psychological, and Spiritual	31	4.8
			Physical, Spiritual, and Capability to carry out daily activities	15	2.3
			Physical, Psychological, and Social	13	2.0
			Physical, Psychological, and Capability to carry out daily activities	13	2.0
			Physical, Psychological, Spiritual, Social, and Economic	11	1.7
			Physical, Psychological, and Healthy Lifestyle	4	0.6
			Physical and Social	3	0.5
			Absence of physical, psychological, and economic problems	2	0.3
Psychological	64	10	Physical and Psychological	99	15.5
			Physical, Psychological, and Spiritual	31	4.8
			Physical, Psychological, and Social	13	2.0
			Physical, Psychological, and Capability to carry out daily activities	13	2.0
			Physical, Psychological, Spiritual, Social, and Economic	11	1.7
			Psychological and Capability to carry out daily activities	10	1.6
			Physical, Psychological, and Healthy Lifestyle	4	0.6
			Absence of physical, psychological, and economic problems	2	0.3
Spiritual	15	2.3	Physical and Spiritual	71	11.1
			Physical, Psychological, and Spiritual	31	4.8
			Physical, Spiritual, and Capability to do daily activities	15	2.3
			Physical, Psychological, Spiritual, Social, and Economic	11	1.7
Capability to carry out daily activities	50	7.8	Physical and Capability to carry out daily activities	44	6.9
			Physical, Spiritual, and Capability to carry out daily activities	15	2.3
			Physical, Psychological, and Capability to carry out daily activities	13	2.0
			Psychological and Capability to carry out daily activities	10	1.6
Healthy lifestyles	35	5.5	Physical, Psychological, and Healthy Lifestyle	4	0.6
Economic *	8	1.3	Absence of physical, psychological, and economic problems	2	1.3

* See Appendix D for sentence examples.

**Table 3:** Themes Regarding the Meaning of Sickness.

Main Themes	*N*	%	Subcategories	*N*	%
Single-Term			In Combination		
Physical	168	26.3	Physical and Psychological	100	15.6
			Physical and Inability to carry out daily activities	59	9.2
			Physical and Spiritual	41	6.4
			Physical, Psychological, and Spiritual	21	3.3
			Physical, Psychological, and Inability to carry out daily activities	13	2.0
			Physical, Psychological, Spiritual, Social, Economic, and Inability to carry out daily activities	12	1.9
			Physical, Psychological, and Social	10	1.6
			Physical and Unhealthy Lifestyle	7	1.1
			Physical and Social	2	0.3
Psychological	43	6.7	Physical and Psychological	100	15.6
			Physical, Psychological, and Spiritual	21	3.3
			Psychological and Activities	15	2.3
			Physical, Psychological, and Inability to carry out daily activities	13	2.0
			Physical, Psychological, Spiritual, Social, Economic, and Inability to carry out daily activities	12	1.9
			Physical, Psychological, and Social	10	1.6
Abnormal circumstances and bad situations	62	9.7			
Spiritual	12	1.9	Physical and Spiritual	41	6.4
			Physical, Psychological, and Spiritual	21	3.3
			Physical, Psychological, Spiritual, Social, Economic, and Inability to carry out daily activities	12	1.9
Inability to carry out daily activities	45	7.0	Physical and Inability to carry out daily activities	59	9.2
			Psychological and Activities	15	2.3
			Physical, Psychological, and Inability to carry out daily activities	13	2.0
			Physical, Psychological, Spiritual, Social, Economic, and Inability to carry out daily activities	12	1.9
Unhealthy Lifestyle **	21	3.3	Physical and Unhealthy Lifestyle	7	1.1
Economic ***	9	1.4	Physical, Psychological, Spiritual, Social, Economic, and Inability to carry out daily activities	12	1.9

** See Appendix E for sentence examples; *** See Appendix F for sentence examples.

## Appendix A. Coding Rules for ‘Single-Term’ and ‘In Combination’ Categories

### Appendix A.1. Single-Term Category

Single-Term category means that respondents perceived their health and/or sickness based on one single condition of health/sickness, so that the statement could be put under one category. If respondents mentioned more than two conditions that could be included in one category, the statement was coded as one answer in one category.

### Appendix A.2. In Combination Category

In combination category means that the respondents’ statements were based on many conditions of health/sickness that could be allocated to two or more categories. If respondents mentioned more than one theme in their answer, the response was recorded as In Combination categories. As a consequence, the number of responses exceeds the number of respondents.

## Appendix B. Coding Rules to Make Categories in Relation to the Meaning of Health

### Appendix B.1. Physical Condition

This category includes responses that mentioned body, body-parts, physical condition, organs, immune system (including hormones and antibodies), having good stamina, being pain-free, being free from bacteria, viruses, and disease.

### Appendix B.2. Psychological Condition

This category includes answers that described psychological condition, including feelings (for example, being comfortable, happy, jubilant, fresh, calm, happy, peaceful, joyful), being sober [sane], free of stress, safe, free of problems, without any burden, in a good situation, and free of suffering.

### Appendix B.3. Spiritual Condition

This category includes statements that mentioned spirit, spiritual, soul, God, faith, and blessing.

### Appendix B.4. Capability to carry out Daily Activities

This category includes sentences that referred to an activity (or activities)/a daily activity (or activities), capability/ability to carry out a daily activity (or activities), ability to do something such as work, have a job, and to be productive.

### Appendix B.5. Healthy Lifestyles

This category includes comments that referred to a healthy lifestyle, healthy diet, dietary habits (including eating and drinking habits), exercise, sleep, and a clean environment.

## Appendix C. Coding Rules to Make Categories in Relation to the Meaning of Sickness

### Appendix C.1. Physical Condition

This category includes responses that referred to body, body-parts, organs, physical condition, immune system, bacteria, viruses, physical disease, pain, stamina, hormones, and antibodies.

### Appendix C.2. Psychological Condition

This category includes answers that referred to psychological condition, mental disorders, feelings (including sadness, suffering, distress), thoughts and problems (including perception, having a lot of things one one’s mind, not being able to find a way out), feeling unsafe, and life burden.

### Appendix C.3. Abnormal Circumstances and Bad Situations

This category includes statements that signified bad situations or abnormal circumstances; something that did not work properly, did not occur as usual, was not normal, or was out of the ordinary.

### Appendix C.4. Spiritual Condition

This category includes comments that mentioned spirit, spiritual, soul, God, temptation, curse, and sickness occurring as the result of sin.

### Appendix C.5. Inability to Carry out Daily Activities

This category includes sentences that indicated activity (or activities), daily activity (or activities) inability to carry out daily activities, or being distracted when performing daily activities inability to work, or being distracted when doing something such as work, not being productive.

## Appendix D. Sentence Examples for Economic Single Term of Health

*‘Invaluable treasure’ (male, 20 years old)*

*‘Most valuable treasure’ (male, 55 years old)*

*‘A lot of money’ (male, 19 years old)*

*‘The most beautiful treasure’ (female 19 years old)*

*‘An expensive thing and must be maintained’ (female 19 years old)*

## Appendix E. Sentence Examples for Unhealthy Lifestyle Single Term of Sickness

*‘When we consume foods that cannot gives [contribute to] health to our body, like fried foods, etc.’ (female, 22 years old)*

*‘Dirty environment, lack of cleanliness’ (female, 45 years old)*

*‘A situation or event that suffered or experienced when we did not live healthy (careless and arbitrary)’ (female, 19 years old)*

*‘Situation where self cannot live healthy and do not take care of own health’ (female, 20 years old)*

*‘Lack of exercise, less clean or dirty environment and less regular sleep, and can cause illness or lack of drinking water’ (male, 26 years old)*

*‘Bad lifestyle’ (male, 18 years old)*

*‘As a result of unable [to] keep the diet, irregular sleep, etc.’ (male, 21 years old)*

## Appendix F. Sentence Examples for Economic Single Term of Sickness

*‘Adverse conditions so we have to pay something’ (male, 27 years old)*

*‘Abnormal circumstances [that happened] to someone which incur losses’ (male, 19 years old)*

*‘No money’ (male, 19 years old)*

*‘Expensive’ (female, 28 years old)*

*‘Non-productive condition’ (female, 28 years old)*

*‘Biggest losses’ (female, 18 years old)*
